# TNAP and EHD1 Are Over-Expressed in Bovine Brain Capillary Endothelial Cells after the Re-Induction of Blood-Brain Barrier Properties

**DOI:** 10.1371/journal.pone.0048428

**Published:** 2012-10-31

**Authors:** Barbara Deracinois, Sophie Duban-Deweer, Gwënaël Pottiez, Roméo Cecchelli, Yannis Karamanos, Christophe Flahaut

**Affiliations:** 1 Université Lille Nord de France, Lille, France; 2 Université d’Artois, LBHE, Lens, France; 3 IMPRT-IFR114, Lille, France; University of Nebraska Medical Center, United States of America

## Abstract

Although the physiological properties of the blood-brain barrier (BBB) are relatively well known, the phenotype of the component brain capillary endothelial cells (BCECs) has yet to be described in detail. Likewise, the molecular mechanisms that govern the establishment and maintenance of the BBB are largely unknown. Proteomics can be used to assess quantitative changes in protein levels and identify proteins involved in the molecular pathways responsible for cellular differentiation. Using the well-established *in vitro* BBB model developed in our laboratory, we performed a differential nano-LC MALDI-TOF/TOF-MS study of Triton X-100-soluble protein species from bovine BCECs displaying either limited BBB functions or BBB functions re-induced by glial cells. Due to the heterogeneity of the crude extract, we increased identification yields by applying a repeatable, reproducible fractionation process based on the proteins' relative hydrophobicity. We present proteomic and biochemical evidence to show that tissue non-specific alkaline phosphatase (TNAP) and Eps15 homology domain-containing protein 1(EDH1) are over-expressed by bovine BCECs after the re-induction of BBB properties. We discuss the impact of these findings on current knowledge of endothelial and BBB permeability.

## Introduction

Over the last decade, it has become clear that the blood-brain barrier (BBB) has a role in a large number of diseases. The BBB is now considered to be an active partner or prime participant [1], [2] (rather than a passive target) in diseases such as obesity, Alzheimer’s disease, multiple sclerosis, stroke, brain cancer and diabetes mellitus. Therapeutic research has identified three distinct aspects, depending on the disease in question: (i) selective, transient disruption of the BBB, (ii) the ability to enable a drug to cross the BBB and, in contrast, (iii) the need to stop BBB leakage [3], [4].

The morphology and functional properties of the brain capillary endothelial cells (BCECs) that form (with other cells) the BBB are now well documented: a decrease in endothelial permeability, fewer caveolae, the reinforcement of tight junctions, fewer pinocytic vesicles, an increase in the number of mitochondria and a higher transendothelial electrical resistance [5], [6]. Large-scale, directed genomics studies (based on comparative analyses of gene expression catalogues or suppression subtractive hybridization) have provided information on tissue-specific gene expression patterns [7]–[12]. A genomic comparison of *in vivo* and *in vitro* brain microvascular endothelial cells (ECs) that de-differentiate in culture yielded a functionally diverse set of 10 genes, the expression of which correlated with a barrier phenotype [13]. Recently, a comparative *in vivo* analysis of the transcription of more than 85 BBB-associated genes showed an overlap in the normal *in situ* expression of these genes along the cerebral vascular tree [14]. Nevertheless, cerebral capillaries preferentially express a number of solute-transport-related genes, whereas cerebral venules tend to express inflammation-related genes. Quantitative PCR profiling of RNA samples from laser capture microdissected microvessels revealed that five membrane protein transcripts (out of 30 selected transcripts) were BBB-specific [15]. Identification of membrane proteins expressed in BBBs could help us to better understand the molecular mechanisms responsible for the barrier's function. Furthermore, selectively expressed proteins may be targets for BBB-related therapeutics.

Concomitantly, recent progress in large-scale and/or differential identification proteomics techniques has generated information on the molecular features of the BCECs [16]–[22]. The quantification of around 30 mouse plasma membrane proteins was reported in 2008 [23]. This study was followed by the quantitative identification of 114 plasma membrane proteins (transporters and receptors) from human brain microvessels [24].

However, although several glial-produced inductive factors or cellular signalling pathways have been identified in the crosstalk between glial cells and BCECs, the fundamental molecular mechanisms that underlie the establishment and maintenance of this phenotype within BCECs remain misunderstood. Crosstalk between BCECs and astrocytes was long time regarded as the main cellular influence on induction of a BBB phenotype; but, there is now a growing body of evidence to suggest that integrated brain function and dysfunction arise from complex interactions between many different cell types [25], [26].

In order to gain a deeper understanding of BBB-related molecular features, we initiated a non-directed, comparative proteomics approach in order to identify proteins potentially involved in the establishment and maintenance of barrier function in the *in vitro* model developed in our laboratory. The complexity of the crude extract of Triton X-100 solubilized proteins from BCECs prevented efficient mass spectrometry (MS) fragmentation analysis and thus the identification of individual proteins. We therefore decided to apply a fractionation process based on the proteins' relative hydrophobicity [22] and demonstrated its repeatability and reproducibility (data not shown). A comparative, off-line, nano-LC MALDI-TOF/TOF-MS analysis enabled the identification of 436 and 408 proteins in bovine BCECs with limited BBB functions ("Lim. BBB", after solo-culture) and re-induced BBB functions ("Re-ind. BBB", after co-culture with glial cells), respectively. Eleven of these (ranging from proteins associated with assembly and organization of the cytoskeleton [21] to those involved in vesicular transport and nucleic acid binding) appeared to be more abundantly in the cytoplasm of Re-ind. BBB cells. We present proteomic and biochemical evidence to suggest that tissue non-specific alkaline phosphatase (TNAP) and Eps15 homology domain-containing protein 1 (EHD1) are over-expressed at the mRNA and protein levels in Re-ind. BBB cells and that this over-expression is accompanied by an increase in alkaline phosphatase (AP) enzymatic activity detected in the cells. Moreover, we found that endothelial permeability was significantly greater when AP activity was specifically inhibited with levamisole - suggesting that TNAP is involved in the regulation of endothelial permeability.

## Experimental Section

### Ethics Statement

All methods involving animal cells were approved by the Direction Départementale des services vétérinaires du Pas-de-Calais (approval #B62-498-5). Brain capillary endothelial cells were isolated from the brain of 6-month-old calves purchased from a local slaughterhouse (Douai, France) while the rats (strain Spragues-Dawley Rjhan) come from Janvier (Le Genest-st-Isle, France).

### Materials

Heat-inactivated calf serum, fetal calf serum, horse serum and DMEM were purchased from GIBCO (Invitrogen Corporation, Carlsbad, CA, USA). L-glutamine was from Merck Chemicals (Darmstadt, Germany). Gentamycin was purchased from Biochrom AG (Berlin, Germany). Six-well plates, 100 mm Petri Dishes and Transwell™ inserts were obtained from Corning Inc. (New York, USA). Alpha-cyano-4-hydroxycinnamic acid was from Bruker Daltonics (Bremen, Germany). Basic fibroblast growth factor, collagenase, monoclonal anti β-actin antibody and levamisole were supplied by Sigma-Aldrich (St Quentin Fallavier, France). Polyclonal anti-TNAP was from GeneTex (Irvine, USA). A monoclonal anti-EHD1 antibody and the Alkaline Phosphatase Colorimetric Assay Kit were purchased from Abcam (Paris, France). Anti-mouse and anti-rabbit immunoglobulins conjugated to HRP were obtained from Dako (Trappes, France). All other reagents were of analytical or electrophoresis grade.

### Cell Culture and the BBB Model

Primary cultures of mixed glial cells were initiated from new-born rat cerebral cortex, as described by Booher and Sensenbrenner [27]. Briefly, glial cells were cultured in 100 mm Petri Dishes or six-well plates in DMEM supplemented with 10% (v/v) heat-inactivated fetal calf serum, 2 mM glutamine and 50 µg/ml gentamycin. Three weeks after seeding, the confluent glial cell cultures were used for co-cultures. Bovine BCECs were isolated and characterized as described by Méresse *et al.* [28], [29]. Bovine BCECs were cultured for 12 days in the absence of glial cells (i.e. in solo-culture, yielding Lim. BBB functions) or the presence of glial cells (i.e. in co-culture, yielding Re-ind. BBB functions) on a Transwell™ insert (pore size: 0.4 µm) coated with extracellular matrix protein (rat tail collagen) in DMEM supplemented with 10% (v/v) heat-inactivated calf serum, 10% (v/v) horse serum, 2 mM glutamine, 50 µg/ml gentamycin and 1 ng/ml basic fibroblast growth factor. Cells were counted after trypsinization. Immunostaining of integral or associated tight junction proteins (zonulae occludens 1, occludin and claudin-5) was performed as described previously [30].

### Cell Harvesting and Protein Extraction

Twenty-four hours after the addition of fresh medium, the ECs growing on a Transwell™ insert (1.5×10^6^ Lim. BBB BCECs and 2.7×10^6^ cells Re-ind. BBB BCECs) were harvested by treatment with *Clostridium histolyticum* collagenase [21]. Briefly, the cells were treated at 37°C for 45 min with 1.5 ml of a collagenase solution (0.1% w/v). The collected material was rinsed three times in PBS-calcium magnesium free and centrifuged for 10 min at 500×*g*. Cell pellets were lysed in 200 µl of lysis buffer [Tris/HCl 10 mM, EDTA 1 mM, Triton X-100 1% (v/v), 2-mercaptoethanol 0.1% (v/v) and protease inhibitors (Roche Biomoleculars, Meylan, France)], broken up with a Pellet Pestle® homogenizer (Kimble Chase Life Science and Research Products LLC, Vineland, NJ; USA), sonicated (nine 10-second cycles at 30 W) and centrifuged (13500×*g*, 4°C, 1 h). The protein content of the Triton X-100-soluble portion was assayed using Peterson’s method [31]. Supernatants were concentrated, desalted and delipidated by overnight organic precipitation at −20°C.

### Nano-LC MALDI-TOF/TOF-MS Experiments

The biological triplicates of Triton-soluble proteins from the Lim. BBB and Re-ind. BBB culture conditions were each fractionated into five sub-fractions (F0, F25, F50, F75 and F100) with increasing concentrations of acetonitrile (0%, 25%, 50%, 75% and 100%, respectively). Briefly, protein pellets were vigorously vortexed at room temperature during 1 h in a pure H_2_O solution and centrifuged 5 min at 14000×*g*. The withdrawn supernatant then constitutes the fraction F0. This is repeated with a solution of 25% H_2_0/75% acetonitrile (v:v) and so on. The fractions were then subjected to trypsin digestion and the peptide were separated on an U3000 nano-LC system (Dionex-LC-Packings, Sunnyvale, CA, USA). After a standard pre-concentration step (C18 cartridge, 300 µm, 1 mm), the peptide samples were separated on a Acclaim PepMap100, C18 column (75 µm i.d. ×15 cm, 3 µm, 100 Å) using an acetonitrile gradient (from 5% to 18.5% acetonitrile over 20 min, from 18.5% to 63.5% over 140 min, from 63.5% to 86% over 15 min and then 15 min in 86% acetonitrile). The flow was set to 300 nl/min and a total of 380 fractions were automatically collected (one every 30 s) on an AnchorChip™ 600 MALDI target by using a Proteineer™ fraction collector (Bruker Daltonics). Matrix (1.8 µl of 0.33 mg/ml α-cyano-4-hydroxycinnamic acid in acetone: ethanol: 0.1% TFA, 3∶6: 1 v/v/v) was added to each deposit during the collection process. The MS (reflectron mode) and MS/MS (lift mode) measurements were performed off-line in automatic mode on an Ultraflex™ II TOF/TOF mass spectrometer (Bruker Daltonics) running FlexControl™ 3.0 software (Bruker Daltonics). External calibration over the 1,000–3,500 mass range was performed using the [M+H]^+^ mono-isotopic ions from bradykinin 1–7, angiotensin I, angiotensin II, substance P, bombesin and adrenocorticotropic hormone (clips 1–17 and clips 18–39) from a peptide calibration standard kit (Bruker Daltonics). Briefly, each MS spectrum was acquired by accumulating data from 500 laser shots with a 25 kV accelerating voltage, a 26.3 kV reflector voltage and a 160 ns pulsed ion extraction. Peptide fragmentation was driven by Warp LC software 1.1 (Bruker Daltonics), according to the following parameters: signal-to-noise ratio >15, more than 3 MS/MS per spot if the MS signal was available, 0.15 Da of MS tolerance for peak merge and the elimination of peaks that appeared in over 35% of the fractions. Precursor ions were accelerated to 8 kV and selected in a timed ion gate. Metastable ions generated by laser-induced decomposition were further accelerated by 19 kV in the lift cell and their masses were measured in reflectron mode. For precursor and daughter ions, each MS/MS spectrum was produced by accumulating data from 200 and 1,000 laser shots, respectively. Peak lists were generated from MS and MS/MS spectra using Flexanalysis™ 3.0 software (Bruker Daltonics). Proteins were identified on the basis of peptide fragmentation fingerprints, according to published guidelines [32]. Database searches with Mascot 2.2 (Matrix Science Ltd, London, UK) were performed in the UniProtKB/Swiss-Prot 57.13 database via ProteinScape 1.3 (Bruker Daltonics). The mass tolerance was set to 75 ppm for the precursor ions and 0.5 Da for the fragment ions. One missing cleavage site was allowed and variable methionine oxidation was also considered. The relevance of protein identities was judged according to the probability-based molecular weight search score [33] (calculated with p<0.05). The false discovery rate was calculated with the decoy option of the Mascot search engine. LC-MS/MS data are available in the European Bioinformatics Institute’s PRIDE database (under accession numbers 22489 to 22498).

### Bioinformatics Resources and Protein Lists

Protein lists were compared using nwCompare software [34]. All identified proteins were converted into gene names with the database for annotation, visualization and integrated discovery (DAVID) bioinformatics resources [35], prior to sorting in the protein analysis through evolutionary relationships (PANTHER) classification system (www.pantehrdb.org). PANTHER is a resource in which genes have been functionally classified by expert biologists on the basis of published scientific experimental evidence and evolutionary relationships. Proteins are classified into families and subfamilies of shared function, which are then categorized by molecular function and biological process ontology terms [36], [37].

### One-dimensional Polyacrylamide Gel Electrophoresis (1D-PAGE) and Western Blot Analysis

Ten µg of each acetonitrile fraction were separated electrophoretically on a 4–12% Bis-Tris Criterion XT Precast Gel (Bio-Rad), as recommended by the manufacturer (55 min, 200 V). Protein bands were stained with silver nitrate [38] prior to image acquisition. Twenty µg of Triton X-100-soluble proteins were separated as described above and then electrophoretically transferred (75 min at 100 V and 4°C) to 0.45 µm polyvinylidene fluoride membrane (Bio-Rad). Membranes were blocked 1 h in TBS (10 mM TRIS and 100 mM NaCl, pH 7.5) containing 0.1% Tween 20 and 5% skimmed milk, incubated with primary antibody (anti β-actin at 1/10,000, anti-EHD1 at 1/40,000 and anti-TNAP at 1/1,000) in a blocking solution at 4°C (20 min for anti β-actin antibody and overnight for the other antibodies), washed three times and incubated with specific secondary antibody conjugated to HRP (anti-mouse at 1/1,000; anti-rabbit at 1/2,000) for 1 h in a blocking solution. The membranes were then rinsed three times with TBS-Tween. Lastly, the immunoblots were visualized using a chemiluminescent substrate (ECL Plus™ Western Blotting Detection Reagent (GE Healthcare, Amersham Bioscience, Orsay, France)).

### Image Acquisition

Images from 1D-PAGE and Western blot experiments were acquired at 300 dots per inch with a freshly calibrated Umax scanner running Labscan 3.0 software (GE Healthcare). Digitized images were stored in Tagged Image File format. Protein bands were quantified with the TotalLab 100 software (Nonlinear Dynamics, Newcastle upon Tyne, UK). Statistical analysis was performed with PRISM 5 software (GraphPad Software, Inc., La Jolla, CA, USA).

### RNA Extraction and the Reverse Transcriptase-polymerase Chain Reaction (RT-PCR)

Bovine BCECs were lysed using RNeasy Lysis Buffer (Qiagen, Valencia, CA, USA). One BCEC-bearing filter was used for each condition and each experiment was performed in triplicate. Total RNA was extracted using an RNeasy kit (Qiagen), according to the manufacturer's protocol. Single-strand DNA was synthesized from 1 µg of total RNA by reverse transcription with Moloney murine leukemia virus reverse transcriptase (Invitrogen). Primers were custom-synthesized by Invitrogen (listed in additional Table S1). DNA was amplified under specific conditions by using a cycle of 94°C for 3 min, 25 to 35 cycles (depending on the primer) of 94°C for 30 s, the indicated annealing temperature for 45 s, 72°C for 1 min and a final incubation at 72°C for 10 min. The various RT-PCR products were size-resolved by 1–2% agarose gel electrophoresis, revealed with GelRed® nucleic acid gel stain (Interchim, Montluçon, France) and visualized using a Gel Doc™ XR device (Bio-Rad). Quantification was carried out with Quantity One software (Bio-Rad) and statistical analysis was performed with PRISM 5 software.

### Levamisole-mediated Inhibition and Alkaline Phosphatase Activity Assays

Twenty-four hours after the addition of fresh medium, glial cells were removed (if necessary) and bovine BCECs were incubated for 4 h at 37°C in culture medium supplemented with various concentrations of levamisole (0 mM (the control condition), 1 mM and 5 mM). Transendothelial permeability was then assessed by measuring the kinetics of clearance of 50 µM Lucifer Yellow (LY) dilithium from the luminal compartment [30]. The transport of LY across the BCECs monolayer was expressed as a permeability coefficient (Pe^LY^, in cm/min). The AP activity was quantified with an Alkaline Phosphatase Colorimetric Assay Kit (Abcam). Endothelial cell death was assessed using the CytoTox-ONE™ Membrane Integrity Assay (Promega Corporation, USA). The extent of cell death in each experimental condition was expressed as percentage of full kill (where the latter corresponded to cells lysed with 9% Triton X-100 (w/v) buffer).

## Results

### Confirmation of BBB-like Properties

**Figure 1:**
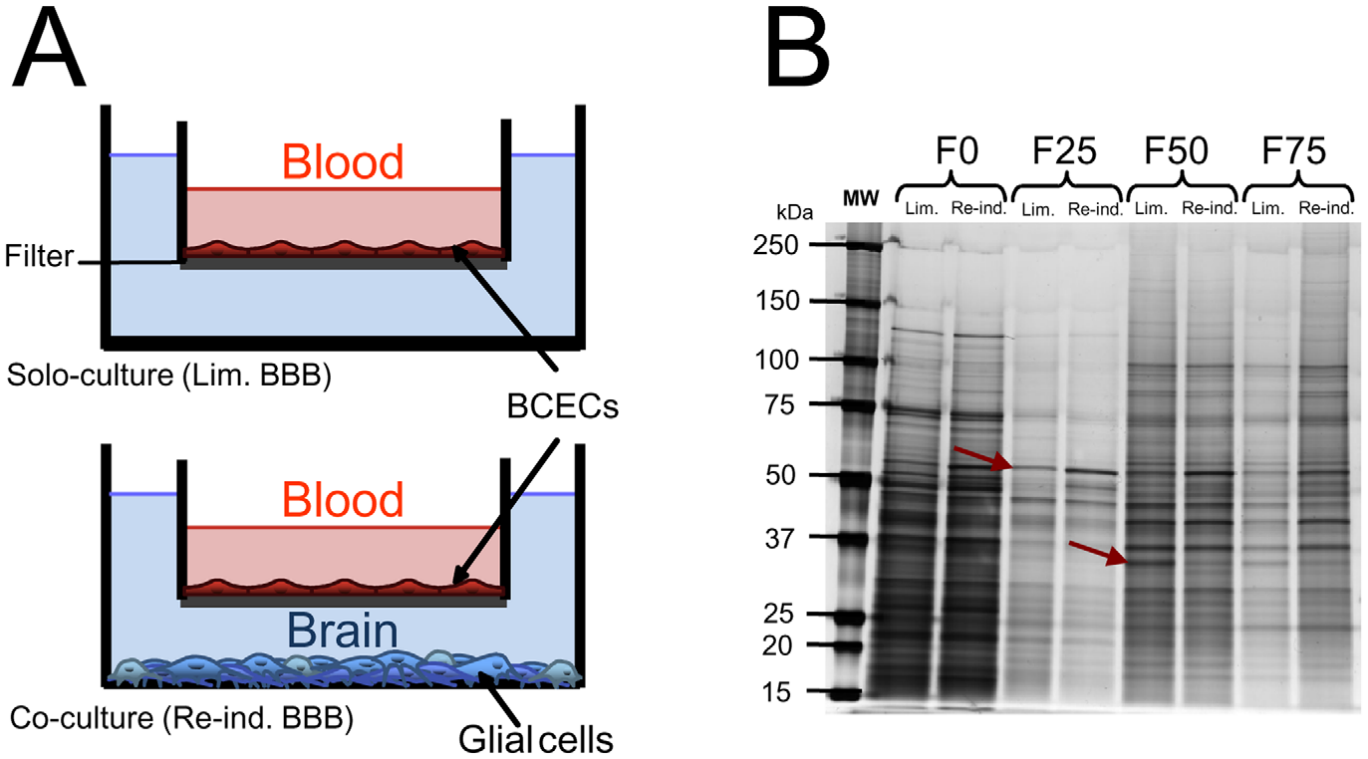
*In vitro* BBB model and assessment of protein fractionation. (A) A schematic drawing of the culture systems used in this study. (B) Gel image from silver-nitrate-stained 1D-PAGE of fractionated Triton X-100-extracted proteins of bovine brain capillary endothelial cells (BCECs) with either limited BBB functions (Lim. BBB) or re-induced BBB functions (Re-ind. BBB). Red arrows correspond to examples of proteins expressed differentially in the two conditions.

As often reported in the literature [39], primary capillary ECs dedifferentiate after isolation *in vitro* and lose their BBB properties. The cells' barrier properties were restored by a 12-day co-culture (Figure 1A) in which bovine BCECs were plated on the upper side of a filter placed in a Petri dish containing glial cells. Re-induction of BBB properties was confirmed by the fact that the transendothelial Pe^LY^ was almost two-fold higher for Lim. BBB bovine BCECs (0.8×10^−3^ cm/min) than for Re-ind. BBB cells (0.4×10^−3^ cm/min). Immunostaining also confirmed the presence and localization of the main tight junction proteins (occludin and claudin-5) and the associated intracellular scaffolding protein zonulae occludens 1, as described previously [30], [40]. Concomitantly, cell numbers per area were about 1.8 fold higher for Re-ind. BBB BCECS than for Lim. BBB BCECS.

### Protein Extraction and Fractionation

Bovine BCECs were harvested by collagenase treatment and then lysed with Triton X-100 buffer [21]. The crude Triton X-100 extract was then subjected to in-solution trypsin proteolysis and then off-line nano-LC MALDI-TOF/TOF-MS analysis. Around 12,000 compounds were detected by MS over the 3 h chromatography run. Unfortunately, the complexity of the samples abolished effective MS/MS fragmentation and only a few proteins were identified (data not shown). The crude extract was therefore divided into the five fractions F0, F25, F50, F75 and F100. As expected on the basis of previous reports [22], the 100% acetonitrile fraction primarily contained the ten major cytoskeletal proteins and was not studied further.

### Assessment of Protein Fractionation

A triplicate protein assay was performed on fractions F0 to F75. The protein concentrations ranged from 2 µg/µl to just below 0.1 µg/µl (±10%). There were no significant differences between homologous fractions, showing that each fraction was equivalent in terms of the amount of protein (data not shown).

The fractions' homogeneity was evaluated by 1D-PAGE and silver nitrate staining (Fig. 1B). The separation profiles showed that the nature of the extracted proteins differed from one fraction to another (with the possible exception of fractions F50 and F75, which has very similar separation profiles). Moreover, the separation profiles for a given fraction were similar under Lim. BBB and Re-ind. BBB conditions. We also observed a lower amount of high-molecular-mass proteins in fraction F25.

### Overall Evaluation of Protein Identities

**Figure 2:**
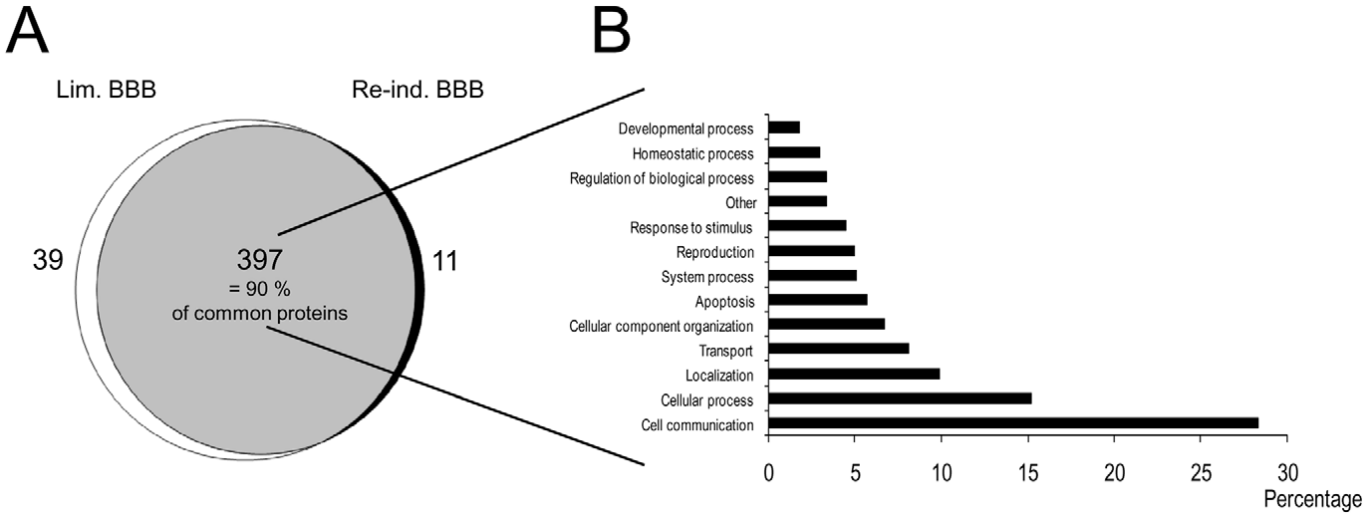
Overall evaluation of protein identities. (A) A Venn diagram showing the common proteins identified in bovine BCECs with limited BBB functions (Lim. BBB) or re-induced BBB functions (Re-ind. BBB) and the proteins identified only in each type of BCEC preparation. (B) Classification by biological processes of the proteins (as identified by nano-LC MALDI-TOF/TOF-MS) found in both BCEC preparations. After conversion to the homologous human genes, proteins were classified using the PANTHER classification system (http://www.pantherdb.org).

After in-solution proteolysis, each fraction was then subjected to a nano-LC MALDI-TOF/TOF-MS analysis. Overall, 447 proteins were identified (Fig. 2A) at least twice and had at least one sequenced peptide (LC-MS/MS data are available in the European Bioinformatics Institute’s PRIDE database under accession numbers 22489 to 22498). In average, the false discovery rate was of 3% ±0.87. The computer-assisted, comparative analysis with the nwCompare algorithm [34] revealed that 397 proteins (90%) were common to both BCECs culture conditions, whereas 39 proteins were only identified in Lim. BBB cell extracts and 11 were only identified in Re-ind. BBB cell extracts. Classification of commonly identified proteins according to the biological processes defined by gene ontology (GO) in PANTHER (Fig. 2B) revealed that almost 45% were involved in cell communication and cellular processes (GO:0007154, ≈28%; GO:0009987, ≈15%, respectively). Interestingly, about 20% of the identified proteins were involved in molecular localization (GO:0051179, ≈10%) and molecular transport (GO:0006810, ≈8%). The fifth category (in terms of decreasing abundance of identified proteins) concerned proteins involved in cellular component organization (GO:0016043, ≈7%). Other GO categories (ranging from apoptosis, response to stimulus and regulation of biological process to homeostatic processes) accounted for between 3% and 6% of the remaining proteins.

The PANTHER classification of 39 condition-specific proteins identified in the Lim. BBB BCECs showed that approximately 75% of the proteins were involved in metabolic processes, cellular processes, transport, developmental processes, cellular component organization and cell communication (data not shown).

### Reverse Transcription PCR and Immunoblotting: Confirmation of Protein-level Changes

**Figure 3:**
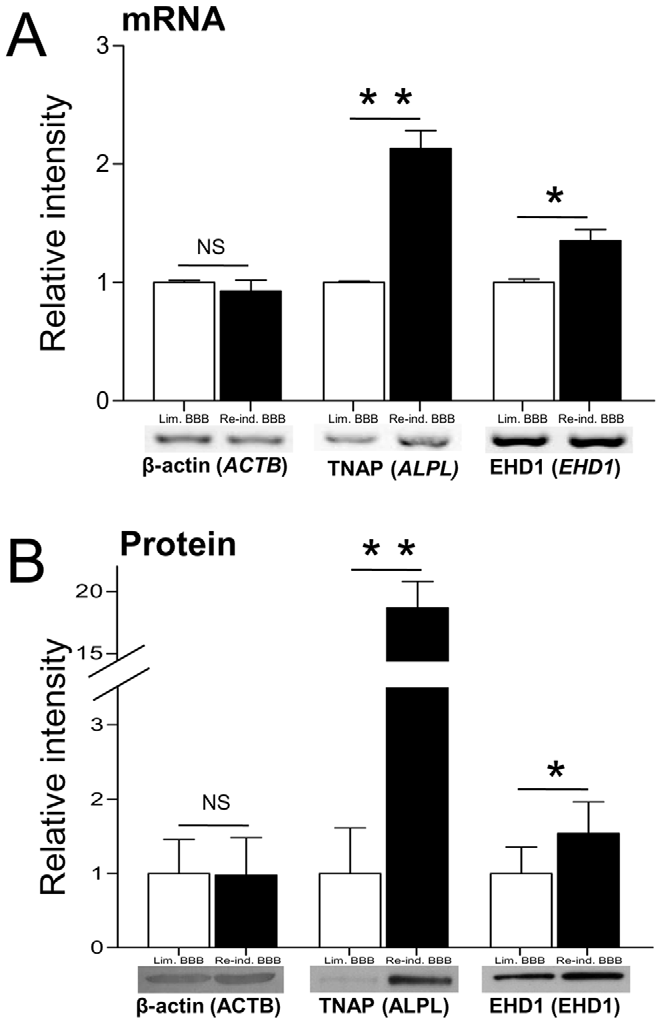
RT-PCR and immunoblotting: confirmation of quantitative changes. (A) RT-PCR analysis: β-actin (*ACTB*), TNAP (*ALPL*) and EHD1 (*EHD1*) mRNA expression detected by RT-PCR analysis from bovine BCECs with limited BBB functions (Lim. BBB) or re-induced BBB functions (Re-ind. BBB). Analysis was performed using the primers and conditions described. (B) Western blot analysis: β-actin (ACTB), TNAP (ALPL) and EHD1 (EHD1) protein levels in Triton X-100 extracts from bovine BCECs. The Western blot analysis was performed using the antibodies and conditions described. Quantitative and statistical analyses were performed with Quantity One (RT-PCR) or TotalLab 100 (Western blots) and PRISM 5 software packages, respectively. The results correspond to the mean ± SEM from three distinct assays. * p<0.03; ** p<0.002; NS: non-significant (in an unpaired t-test for RT-PCR and a paired t-test for Western blots). The expression of β-actin was monitored as a sample quality control.

Table 1 summarizes the identities of proteins identified only in one kind of BCEC culture and not in the other. Of the 11 condition-specific proteins identified in Re-ind. BBB BCECs, tissue non-specific alkaline phosphatase (TNAP) and Eps15 homology domain (EHD)-containing protein 1 (EHD1) were studied in more detail because of their importance in BBB and vesicular trafficking, respectively. An RT-PCR analysis of mRNA transcript levels (Fig. 3A) demonstrates that the expression levels of TNAP (*ALPL*) and EHD1 (*EHD1*) mRNA transcripts are expressed significantly more strongly in Re-ind. BBB cells than in Lim. BBB cells (+100% for *ALPL* and +30% for *EHD1*) when compared to the unchanged expression level of the β-actin mRNA transcript.

**Table 1:** List of proteins identified (in at least two out of three nano-LC MALDI-TOF/TOF-MS analyses and at least one peptide sequenced) in one type of BCECs and not in the other.

Protein Name	Swiss-Prot Accesion	Gene Name	Accession Number	Molecular Weight (Da)	Isoelectric Point	Identification Score^a^	Sequence Coverage (%)	Number of fragmented peptides	Number of fraction	Identified only in^b^
Abhydrolase domain-containing protein 14B	ABHEB_BOVIN	ABHD14B	A7YY28	22 441	6,05	43,9	13	2	2	Re-ind. BBB
ADP-ribosylation factor 4	ARF4_BOVIN	ARF4	Q3SZF2	20 515	5,91	159,7	20,11	2	2	Re-ind. BBB
EH domain-containing protein 1	EHD1_BOVIN	EHD1	Q5E9R3	60 644	6,4	75,7	4,66	2	2	Re-ind. BBB
Dolichyl-diphosphooligosaccharide–protein glycosyltransferase 48 kDa subunit	OST48_BOVIN	DDOST	A6QPY0	48 761	5,52	40,7	3,41	1	2	Re-ind. BBB
Alkaline phosphatase, tissue-nonspecific isozyme	PPBT_BOVIN	ALPL	P09487	57 156	6,28	42,5	6,1	2	2	Re-ind. BBB
SUMO-activating enzyme subunit 1	SAE1_BOVIN	SAE1	A2VE14	38 281	5,15	84,3	2,77	1	2	Re-ind. BBB
Splicing factor, arginine/serine-rich 9	SFRS9_RAT	SFRS9	Q5PPI1	25 482	8,67	33,5	5,42	1	2	Re-ind. BBB
Structural maintenance of chromosomes flexible hinge domain-containing protein 1	SMHD1_MOUSE	SMCHD1	Q6P5D8	225 506	6,87	41,2	2,7	2	2	Re-ind. BBB
Thioredoxin-related transmembrane protein 2	TMX2_BOVIN	TMX2	Q2TBU2	34 007	8,89	89,4	5	1	2	Re-ind. BBB
Tropomyosin alpha-1 chain	TPM1_BOVIN	TPM1	Q5KR49	32 675	4,69	48,1	2,77	1	3	Re-ind. BBB
Xanthine dehydrogenase/oxidase	XDH_BOVIN	XDH	P80457	146 696	7,97	116,5	3,23	3	3	Re-ind. BBB
26S protease regulatory subunit 6A	PRS6A_RAT	Psmc3	Q63569	49 129	5,13	45,1	3,64	1	2	Lim. BBB
Class I histocompatibility antigen, GOGO-A0101 alpha chain	1A01_GORGO		P30375	40 804	5,91	191,1	6,57	2	2	Lim. BBB
Abhydrolase domain-containing protein 11	ABHDB_BOVIN	ABHD11	Q3SZ73	33 527	9,55	46,7	6	1	3	Lim. BBB
Serum albumin	ALBU_BOVIN	ALB	P02769	69 248	5,82	105,9	5,1	2	3	Lim. BBB
Beta-2-microglobulin	B2MG_BOVIN	B2M	P01888	13 668	7,79	114,8	21,18	2	3	Lim. BBB
Carbohydrate sulfotransferase 7	CHST7_RAT	Chst7	Q6XQG8	55 057	9,9	33,4	2,47	1	2	Lim. BBB
Cytochrome c oxidase subunit 4 isoform 1, mitochondrial	COX41_BOVIN	COX4I1	P00423	19 559	9,32	32,5	7,1	1	2	Lim. BBB
Copine-6	CPNE6_BOVIN	CPNE6	O95741	61 952	5,32	53,9	5,38	2	2	Lim. BBB
COP9 signalosome complex subunit 8	CSN8_RAT	Cops8	Q6P4Z9	23 221	5,09	52,1	17,22	2	2	Lim. BBB
Drebrin	DREB_RAT	DBN1	Q07266	77 424	4,46	66	6	2	3	Lim. BBB
Clathrin interactor 1	EPN4_BOVIN	CLINT1	A7Z035	70 477	6,15	49,1	1	1	3	Lim. BBB
Serine hydroxymethyltransferase, mitochondrial	GLYM_BOVIN	SHMT2	Q3SZ20	55 570	7,62	73	7,53	2	2	Lim. BBB
Heterogeneous nuclear ribonucleoprotein R	HNRPR_HUMAN	HNRNPR	O43390	70 899	8,23	49,1	3,47	2	5	Lim. BBB
Isocitrate dehydrogenase [NADP], mitochondrial	IDHP_BOVIN	IDH2	Q04467	50 707	8,88	51,6	4,86	2	3	Lim. BBB
Protein KIAA0284	K0284_MOUSE	Kiaa0284	Q80U49	170 718	6,42	34,8	2,7	2	2	Lim. BBB
UMP-CMP kinase	KCY_BOVIN	CMPK1	Q2KIW9	22 265	5,66	55,5	6,12	1	2	Lim. BBB
U6 snRNA-associated Sm-like protein LSm7	LSM7_HUMAN	LSM7	Q9UK45	11 595	5,1	72,9	24,27	2	2	Lim. BBB
Microtubule-associated protein 4	MAP4_BOVIN	MAP4	P36225	111 846	4,85	73,7	2,14	1	2	Lim. BBB
Malignant fibrous histiocytoma-amplified sequence 1	MFHA1_HUMAN	MFHAS1	Q9Y4C4	116 838	8,02	40,8	2,7	2	2	Lim. BBB
Myoferlin	MYOF_HUMAN	MYOF	Q9NZM1	233 177	5,83	95,3	0,92	1	2	Lim. BBB
NHL repeat-containing protein 3	NHLC3_MOUSE	NHLRC3	Q8CCH2	38 171	5,81	47,8	3	1	2	Lim. BBB
Protein NipSnap homolog 2	NIPS2_HUMAN	GBAS	O75323	33 721	9,42	53,9	6	1	2	Lim. BBB
Prolyl 4-hydroxylase subunit alpha-2	P4HA2_MOUSE	P4HA2	Q60716	60 964	5,55	175,8	13,96	5	8	Lim. BBB
Delta-1-pyrroline-5-carboxylate synthetase	P5CS_HUMAN	ALDH18A1	P54886	87 248	6,66	47,2	1,5	1	3	Lim. BBB
Lysophosphatidylcholine acyltransferase 2-B	PCATB_RAT	Aytl1b	Q4V8A1	58 526	8,71	45,2	3	2	2	Lim. BBB
Protein disulfide-isomerase A4	PDIA4_BOVIN	PDIA4	Q29RV1	72 481	4,99	50	2,79	1	3	Lim. BBB
PDZ and LIM domain protein 5	PDLI5_RAT	PDLIM5	Q62920	63 161	8,73	48,9	1,86	1	3	Lim. BBB
Ribose-phosphate pyrophosphokinase 1	PRPS1_BOVIN	PRPS2	Q2HJ58	34 812	6,51	91,5	2,7	1	2	Lim. BBB
26S protease regulatory subunit S10B	PRS10_BOVIN	PSMC6	Q2KIW6	44 046	6,74	42	5,4	2	2	Lim. BBB
Ras-related protein Rab-2A	RAB2A_RAT	RAB2A	P05712	23 521	6,08	31,2	6,13	1	2	Lim. BBB
RNA-binding protein Raly	RALY_MOUSE	RALY	Q64012	33 138	8,94	39,8	3,52	1	2	Lim. BBB
40S ribosomal protein S14	RS14_RAT	Rps14	P13471	16 249	10,07	96,2	14	1	2	Lim. BBB
Splicing factor 1	SF01_HUMAN	SF1	Q15637	68 286	9,07	67,4	5,55	1	2	Lim. BBB
Sideroflexin-3	SFXN3_BOVIN	SFXN3	A6QP55	35 685	9,66	81,2	9	2	3	Lim. BBB
Small nuclear ribonucleoprotein Sm D2	SMD2_BOVIN	SNRPD2	Q3SZF8	13 518	9,92	78,8	16,1	1	2	Lim. BBB
Threonyl-tRNA synthetase, cytoplasmic	SYTC_BOVIN	TARS	Q3ZBV8	83 439	6,34	62,7	2,9	2	2	Lim. BBB
Transforming growth factor-beta receptor-associated protein 1	TGFA1_BOVIN	TGFBRAP1	Q8WUH2	97 096	6,1	36	2,7	2	2	Lim. BBB
Tax1-binding protein 3	TX1B3_MOUSE	TAX1BP3	Q9DBG9	13 714	8,04	104,5	14,51	1	2	Lim. BBB
Vigilin	VIGLN_RAT	HDLBP	Q9Z1A6	141 496	6,51	49,7	1,65	1	3	Lim. BBB

a Identification score: the relevance of protein identities was judged according to the probability-based molecular weight search score [33] (calculated with p<0.05)). The significance threshold of ions scores corresponds to the value of 31.

b Protein identified in brain capillary endothelial cells displaying limited BBB (Lim. BBB) or re-induced BBB (Re-ind. BBB) functions.

**Figure 4:**
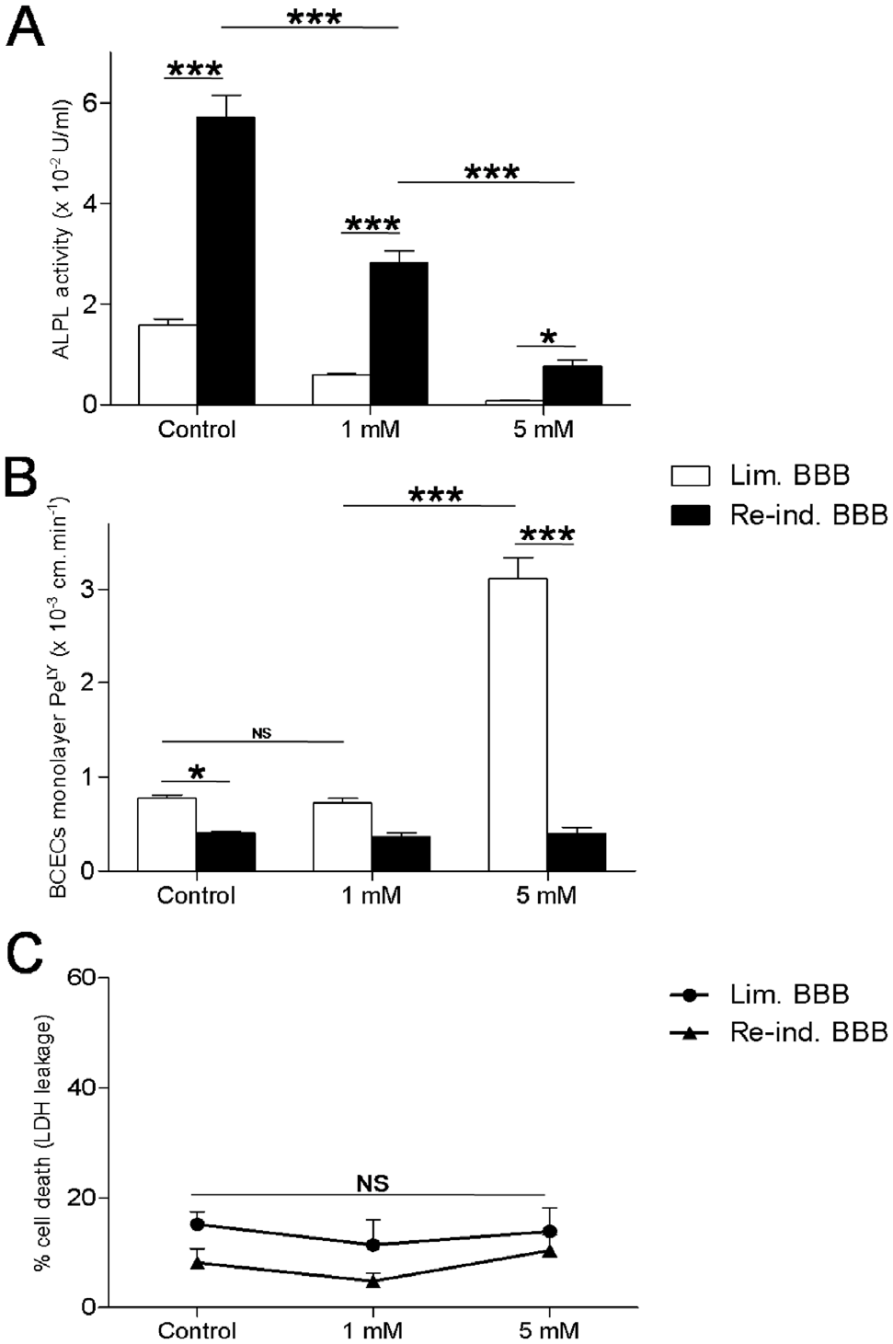
Alkaline phosphatase activity assay and levamisole-mediated inhibition. (A) Alkaline phosphatase activity, (B) monolayer permeability to Lucifer Yellow (Pe^LY^) and (C) percentage of endothelial cell death (LDH leakage into the culture medium): determined after 4h of incubation of bovine BCECs with limited BBB functions (Lim. BBB) or re-induced BBB functions (Re-ind. BBB) with different concentrations of levamisole (0 to 5 mM). The extent of cell death in each experimental condition was expressed as percentage of full kill (where the latter corresponded to cells lysed with 9% Triton X-100 (w/v) buffer). The statistical analysis was carried out using PRISM 5 software. The results correspond to the mean ± SEM (N = 3, n = 9 BCEC monolayers per treatment). The significance of between-experiment differences were tested in a two-way analysis of variance, followed by a Bonferroni *post hoc* test. * p<0.05; *** p<0.001; NS: non-significant.

Concomitantly, expression levels of TNAP and EHD1 proteins were checked by immunoblotting and the AP activity was assayed with a commercially available kit (Fig. 3B and Fig. 4A-Control, respectively). Overall protein expression level of β-actin did not differ significantly in the two conditions (Fig. 4B; negative control). As expected, the protein expression levels (Fig. 3B) of TNAP and EHD1 were greater in Re-ind. BBB BCECs. The statistical significance of the fold-changes affecting TNAP and EHD1 was confirmed in paired t-tests. Moreover, AP activity was 3.5-fold higher (or 2-fold higher, when normalized against the number of cells) in Re-ind. BBB cells than in Lim. BBB cells (Fig. 4A-Control).

### Levamisole-mediated Inhibition

The influence of TNAP activity on endothelial permeability was investigated by applying the uncompetitive AP inhibitor levamisole [41]. Briefly, Lim. BBB and Re-ind. BBB BCECs were separately incubated for 4 h in culture medium supplemented with 0 mM (control), 1 mM and 5 mM levamisole. A lactate-dehydrogenase-based cytotoxicity activity showed that even the highest concentration of levamisole did not significantly induce cell death during this incubation period (Fig. 4C). The AP assay revealed a dose-dependent decrease in phosphate hydrolysis (Fig. 4A), which reached 95% in Lim. BBB and 86% in Re-ind. BBB BCECs when exposed to 5 mM levamisole. Interestingly, the endothelial monolayer permeability (Fig. 4B) was unaffected at levamisole concentrations of 1 mM, whereas a concentration of 5 mM provoked a 4- fold increase in endothelial permeability in Lim. BBB BCECs but not in Re-ind. BBB BCECs. This result suggests that TNAP protects against the permeability increase mediated by levamisole.

## Discussion

Although marked progress has been made over the last decade, the process by which BCECs differentiate to obtain the BBB phenotype is poorly documented in molecular terms. In order to gain a deeper understanding of BBB-related molecular features, we have been applying a range of proteomic tools to our well-characterized *in vitro* BBB model for the last few years. We are particularly seeking to describe the protein abundance changes that occur during the glial cell-driven re-induction of the BBB phenotype in BCECs.

In proteomics, crude extracts of cell proteins are challenging for many reasons: the features of the extracted cell types, the varying abundances of proteins in a cell and the particular properties of hydrophobic proteins. Under these conditions, shotgun approaches can show their limits. We showed that the heterogeneity of a crude Triton X-100-soluble protein extract can be efficiently and reproducibly reduced by organic fractionation before nano-LC MALDI-TOF/TOF-MS analysis. Ninety percent of the 447 identified proteins were identified in protein extracts from both Lim. BBB and Re-ind. BBB BCECs, the phenotypes of which are thus not completely different.

In this differential proteomics study with biochemical and immunological confirmation, we generated evidence to show that TNAP was overexpressed at the mRNA and protein levels in Re-ind. BBB BCECs and that the increase in TNAP enzymatic activity was correlated with protein levels. The AP family (EC. 3.1.3.1) catalyzes the hydrolysis of phosphomonoesters to produce inorganic phosphate and an alcohol from a variety of organic compounds: nucleosides (5′ -tri-, -di- and -mono- phosphates), pyrophosphate inorganic, pyridoxal-5‘-phosphate and phosphorylated proteins [42], [43]. Hence, the APs can control not only cellular ectonucleosidase and protein dephosphorylation activities but also bone mineralization. Notably, astrocytes release ATP and other nucleotides [44]. The APs are dimeric isoenzymes that are bound to the extracellular surface of plasma membrane microdomains by glycosylphosphatidylinositol anchors. In mammalian cells, four isoenzymes have been described and named according to the tissue specificity (or not) of their expression: intestinal alkaline phosphatase, placental alkaline phosphatase, germ cell alkaline phosphatase and liver-type, bone-type or kidney-type TNAP. Expression of the TNAP types depends on alternative transcription initiation processes but results in the same amino acid sequence [45].

Most of the AP expression data in nervous tissue relate to combined enzyme assays and histological studies. The general presence of AP activity in the brain was reported some time ago [46]–[48] but more precise reports concerning the cerebral parenchyma and the BCECs are more recent [49]–[51]. This characteristic was also noted in several *in vitro* models of mammalian BBBs [52]–[54]. Alkaline phosphatase activity has long been used as a brain endothelium marker [55], [56]. In fact, TNAP is the only AP isoform found in the brain [57], [58]. It has been suggested that BCEC maturation during brain development is corroborated with high TNAP activity [59]. Beck *et al*. [60] first mentioned that TNAP activity was markedly elevated when the ECs were co-cultured with glial cells. This fact was recently confirmed [61] by a study showing that bone-type TNAP is present in neurons and brain ECs in several species. The study also revealed strongly down-regulated expression of the bone TNAP transcript when mouse BCECs were maintained in solo-culture for a few days [61]. Interestingly, a variety of molecules are known to modulate AP expression (retinoic acid [62]–[64]; cAMP [65]; glucocorticoids [62], [66]; transforming growth factor-beta [53]; IL-6 [53] and basic fibroblast growth factor [54], [67]) and brain capillary endothelial permeability. In the present study, we found that glial-cell-induced TNAP expression prevented a levamisole-induced increase in endothelial permeability - suggesting a protective effect.

The best-investigated function of TNAP is its involvement in bone mineralization and remodeling [68], although this appears to be out of the scope of the BBB field. However, there are several lines of evidence in favor of an important role for TNAP in brain function. Hypophosphatasia is one of several brain diseases that are related (at least in part) to the BBB [69], [70]. It results from various mutations of the TNAP gene and is associated with neurological disorders such as mental retardation, seizures and epilepsy [71]–[73]. Furthermore, TNAP is thought to be involved in neurotransmitter metabolism and the availability of pyridoxal-5′-phosphate (PLP) in the brain [74]. We hypothesize that TNAP may regulate the extracellular concentration of PLP through dephosphorylation, thus allowing pyridoxine (the dephosphorylated form of PLP) to cross the BBB [75]. Interestingly, alimentary B1 avitaminosis reportedly produces a decline in AP activity in the rat brain [76]. Moreover, an alimentary deficiency of vitamin B6 (a precursor of PLP) promotes epileptic seizures (as observed in TNAP −/− mice) – seizures that can be suppressed by administration of the vitamin [77], [78]. This co-factor is involved in many different reactions (such as the decarboxylation of amino acids) in the production of a variety of molecules, ranging from neurotransmitters to polyamines [79].

It was recently reported that TNAP (in concert with the ionotrophic P2X_7_ ATP receptor) promotes axonal growth of hippocampal neurons by regulating (via its ectonucleosidase activity) the extracellular ATP concentration [80]. *In vitro*, TNAP promotes the neurotoxic effect of extracellular Tau protein [81]. *In vivo*, TNAP activity is found to be elevated in the hippocampus of patients with AD [82]. Since TNAP promotes the expression of the BBB phenotype in BCECs, one can legitimately suppose that reducing the BBB permeability can enhance Tau-mediated brain lesions by reducing cerebral clearance of the Tau protein.

Despite its known phosphomonoesterase activity, TNAP's putative ability to dephosphorylate protein targets remains subject to debate. Direct proof of TNAP's action on the phosphorylation state of lamini has been reported [58] but contrasts with evidence to show that TNAP does not modulate the phosphorylation of plasma membrane proteins [83]. A differential proteomics study investigating the phosphoprotein changes that occur in Lim. BBB and Re-ind. BBB BCECs would probably provide valuable information on how the BBB phenotype is established.

Several researchers have suggested that AP is associated with transport systems [84] in general and organic cation transport in particular [85]–[87]. Here, we found significant, differential expression of the EHD1 at the mRNA transcript and protein levels. The protein is the most extensively studied member (and the archetype) of the Eps15 homology domain (EHD)-containing family. The latter co-controls the endocytic recycling and transport of receptors internalized through clathrin-dependent or -independent endocytic pathways [88]. More specifically, EHD1 is a serum-inducible serine phosphoprotein [89] that is many found in the endocytic recycling compartment [90]. In fact, EHD1 is thought to co-regulate the exit of a wide range of proteins (including the transferrin receptor (TfR) [91], the cystic fibrosis conductance regulator [92], the major histocompatibility complex class I [93], integrin β1 [94], the glucose transporter type 4 [95]) from this compartment to the plasma membrane. Interestingly, the TfR is specifically expressed on the luminal side of BCECs [96] and levels increase in co-culture with glial cells [97]. Indeed, the TfR is at the heart of efforts seeking to deliver drugs to the brain (see [98] for a review). Given that astrocytes and oligodendrocytes lack TfR, these cells could influence iron import by the BCECs [99]. Synthesis of the TfR is regulated at the mRNA level by five iron-responsive elements present in untranslated mRNA regions, leading to the overexpression of the TfR in iron-depleted situations. However, low extracellular iron levels do not prompt greater TfR expression in BCECs, despite considerably higher blood-to-brain iron transport [100]. This observation suggests that iron depletion raises the recycling rate for endosomes containing TfRs. Although the nature of iron transport across BCECs remains subject to debate (with either receptor-mediated endocytosis or receptor-mediated transcytosis; see [99], [101] for a review), it is clear that iron is indeed transported from the blood to the brain and this transport begins by endocytosis of the iron-lactoferrin-TfR complex (after which TfR would recycled to the plasma membrane). Therefore, EHD1 overexpression in Re-ind. BBB BCECs may constitute indirect evidence of faster recycling TfR. Likewise, glucose transporter type 4 and integrin β1 are expressed by the BBB endothelium [102], [103].

It is known that EHD1 can bind directly to a variety of phospholipids [104], [105] and regulate cell cholesterol homeostasis - suggesting its involvement in low density lipoprotein-receptor (LRP1) internalization [106]. LRP1-mediated endocytosis has also been mentioned in connection with the internalization of various molecules at the BBB endothelium. Several other proteins also interact with EHD1, including rabenosyn-5, EH-binding protein 1, syndapins I and II and rab11 family-interacting protein 2 (see [107] for a review).

EHD1 also regulates actin dynamics by interacting with the Rho-family members GTPase Rac 1 and cell division control protein 42 homolog (CDC42) [108], [109]. We have already reported quantitative changes in levels of actin-related proteins in BCECs displaying BBB features [21], [110].

### Conclusion

For the first time, we used our novel fractionation method [22] to perform a differential analysis of Triton X-100-soluble proteins extracted from Lim. BBB and Re-ind. BBB BCECs. We demonstrated that the fractionation method was reliable and reproducible. The proteins highlighted by proteomics were validated by biochemical techniques. We identified 11 proteins (notably including TNAP and EHD1) that were overexpressed in Re-ind. BBB BCECs. The overexpression of TNAP and EHD1 was confirmed by RT-PCR, immunoblotting and (for TNAP) an AP assay, thus corroborating previous studies. Our initial results for EDH1 overexpression suggest that endocytic recycling and transport is probably accelerated in ECs with a barrier phenotype. Various lines of evidence indicate that TNAP is involved in the transport of pyridoxine across the BBB and the compound's subsequent use in neurotransmitter metabolism. We also demonstrated that glial-cell-induced TNAP overexpression prevents the increase in endothelial permeability otherwise induced by the uncompetitive AP inhibitor levamisole. This finding suggests that TNAP may be an important component in the establishment and maintenance of the BBB phenotype in BCECs. However, the nature of TNAP actions (i.e. ectonucleosidase or phosphatase activities or both) and the mechanism underlying the enzyme's involvement in the glial-cell-induced process remain to be determined.

## Supporting Information

Table S1 **DNA primers and conditions used to amplify mRNA.**
(XLSX)
